# The geography of overdose in British Columbia.

**DOI:** 10.23889/ijpds.v7i3.2099

**Published:** 2022-08-25

**Authors:** Amanda Slaunwhite, Kevin Hu, Brian Klinkenberg, Huiyuan Zhang

**Affiliations:** 1 University of British Columbia; 2 BC Centre for Disease Control

Illicit drug toxicity poisoning (overdose) continues to be a public health emergency in British Columbia (BC) with record high rates of illicit drug toxicity during the COVID-19 pandemic. In 2021, 2224 people died of overdose in BC with some of the highest rates recorded in rural and remote regions. Understanding the geographic variations in overdose mortality risk is necessary to avoid disproportionate risk resulting from service access inequity. Using novel linked administrative health data from the BC Provincial Overdose Cohort we estimated the odds of fatal overdose per event (2015 - 2018) using both conventional logistic regression and Generalized Additive Models (GAM). The results of GAM were mapped to identify spatial-temporal trends in the risk of fatal overdose. We found that the likelihood of fatal overdose was about 20% higher in rural areas than in large urban centers, with some regions reporting odds 50% higher than others. Temporal variations in fatal risk exhibit an increasing trend over the entire province. However, risks in the Interior and Northern BC increased earlier and faster. The results of this study demonstrate the importance of geography to health outcomes and suggest that rural and remote regions may lack harm reduction services to counteract the province-wide increase in illicit drug toxicity death.

